# BPR1K653, a Novel Aurora Kinase Inhibitor, Exhibits Potent Anti-Proliferative Activity in MDR1 (P-gp170)-Mediated Multidrug-Resistant Cancer Cells

**DOI:** 10.1371/journal.pone.0023485

**Published:** 2011-08-24

**Authors:** Chun Hei Antonio Cheung, Wen-Hsing Lin, John Tsu-An Hsu, Tzyh-Chyuan Hour, Teng-Kuang Yeh, Shengkai Ko, Tzu-Wen Lien, Mohane Selvaraj Coumar, Jin-Fen Liu, Wen-Yang Lai, Hui-Yi Shiao, Tian-Ren Lee, Hsing-Pang Hsieh, Jang-Yang Chang

**Affiliations:** 1 National Institute of Cancer Research, National Health Research Institutes, Tainan, Taiwan R.O.C.; 2 Institute of Biotechnology and Pharmaceutical Research, National Health Research Institutes, Zhunan, Miaoli County, Taiwan R.O.C.; 3 Institute of Biochemistry, Kaohsiung Medical University, Kaohsiung, Taiwan R.O.C.; 4 Centre for Bioinformatics, School of Life Sciences, Pondicherry University, Kalapet, Puducherry, India; 5 Division of Hematology and Oncology, Department of Internal Medicine, National Cheng Kung University Hospital, Tainan, Taiwan R.O.C.; Enzo Life Sciences, Inc., United States of America

## Abstract

**Background:**

Over-expression of Aurora kinases promotes the tumorigenesis of cells. The aim of this study was to determine the preclinical profile of a novel pan-Aurora kinase inhibitor, BPR1K653, as a candidate for anti-cancer therapy. Since expression of the drug efflux pump, MDR1, reduces the effectiveness of various chemotherapeutic compounds in human cancers, this study also aimed to determine whether the potency of BPR1K653 could be affected by the expression of MDR1 in cancer cells.

**Principal Findings:**

BPR1K653 specifically inhibited the activity of Aurora-A and Aurora-B kinase at low nano-molar concentrations *in vitro*. Anti-proliferative activity of BPR1K653 was evaluated in various human cancer cell lines. Results of the clonogenic assay showed that BPR1K653 was potent in targeting a variety of cancer cell lines regardless of the tissue origin, p53 status, or expression of MDR1. At the cellular level, BPR1K653 induced endo-replication and subsequent apoptosis in both MDR1-negative and MDR1-positive cancer cells. Importantly, it showed potent activity against the growth of xenograft tumors of the human cervical carcinoma KB and KB-derived MDR1-positive KB-VIN10 cells in nude mice. Finally, BPR1K653 also exhibited favorable pharmacokinetic properties in rats.

**Conclusions and Significance:**

BPR1K653 is a novel potent anti-cancer compound, and its potency is not affected by the expression of the multiple drug resistant protein, MDR1, in cancer cells. Therefore, BPR1K653 is a promising anti-cancer compound that has potential for the management of various malignancies, particularly for patients with MDR1-related drug resistance after prolonged chemotherapeutic treatments.

## Introduction

Mitosis is a key step in cell cycle that is tightly regulated by many proteins. Abnormal expression or activation of these regulatory proteins could result in aberrant mitosis, leading to the development of cancers [1], [2]. At the molecular level, Aurora kinases (Aurora-A, Aurora-B and Aurora-C) are serine/threonine kinases that function as key regulators of mitosis. Under normal physiological conditions, they are essential for spindle assembly, centrosome maturation, chromosomal segregation and cytokinesis [3], [4]. Under pathological conditions, it has been demonstrated that Aurora kinases are over-expressed in various human cancers and also played important roles in the process of tumorigenesis [5], [6], [7], [8]. For example, Aurora-A kinase is over-expressed in upper gastrointestinal adenocarcinomas [6]. In addition, a correlation between Aurora-A expression levels and tumor progression has been demonstrated in patients with head and neck squamous cell carcinoma [9]. On the other hand, Aurora-B kinase is frequently over-expressed in primary NSCLC and malignant gliomas, particularly glioblastomas [10], [11]. Since over-expression of Aurora-A and Aurora-B is frequently associated with tumorigenesis, these molecules have been targeted for cancer therapy. The first proof-of-concept pan-Aurora kinase inhibitor, VX-680 (MK-0457, Tozasertib), was developed in 2004 by Vertex Pharmaceuticals (in collaboration with Merck) with an aim to target cancer cells. This specific inhibitor has been shown effective in targeting cancer cells both *in vitro* and *in vivo*, and has received approval from the US Food and Drug Administration (FDA) to enter clinical trials [12], [13], [14]. Since then, continuous efforts have been made by different pharmaceutical companies in search of potential Aurora kinase inhibitors that exhibit better therapeutic profile and specificity as compare to the first generation inhibitor, VX680 [15], [16], [17], [18], [19], [20], [21], [22], [23].

Despite early successes of the development of various Aurora kinase inhibitors, recent studies reveal that the effectiveness of many of these developed and clinically tested inhibitors, including VX680, PHA-739358 and AZD1152, can be affected by the expression of multidrug resistance protein MDR1 (P-gp170) in cancer cells [24], [25]. In fact, over-expression of MDR1 also interferes with a broad range of different chemotherapeutic agents [2], [26], [27], [28], [29]. For examples, expression of the trans-membrane drug efflux pump, MDR1, reduces the sensitivity of cancer cells to paclitaxel, vincristine (anti-microtubule agents), doxorubicin (DNA intercalating agent), mitoxantrone, VP-16 (topoisomerase II inhibitors) and imatinib (tyrosine kinase inhibitor) [28], [30], [31], [32], [33], [34]. Therefore, there has been great interest in identifying novel anti-cancer compounds that can overcome MDR1-related resistance and also exhibit improved pharmacological profiles.

In this study, a novel pan-Aurora kinase inhibitor entitled BPR1K653 was developed and its potency against various MDR1-negative and MDR1-positive cancer cells was evaluated. Results of the current study show that unlike the above mentioned chemotherapeutic agents, BPR1K653 is effective in targeting both MDR1-negative and -positive cancer cells *in vitro* and *in vivo*. Furthermore, BPR1K653 exhibits favorable pharmacokinetic properties *in vivo*.

## Results

### BPR1K653 is a potent and selective pan-Aurora kinase inhibitor

**Figure 1:**
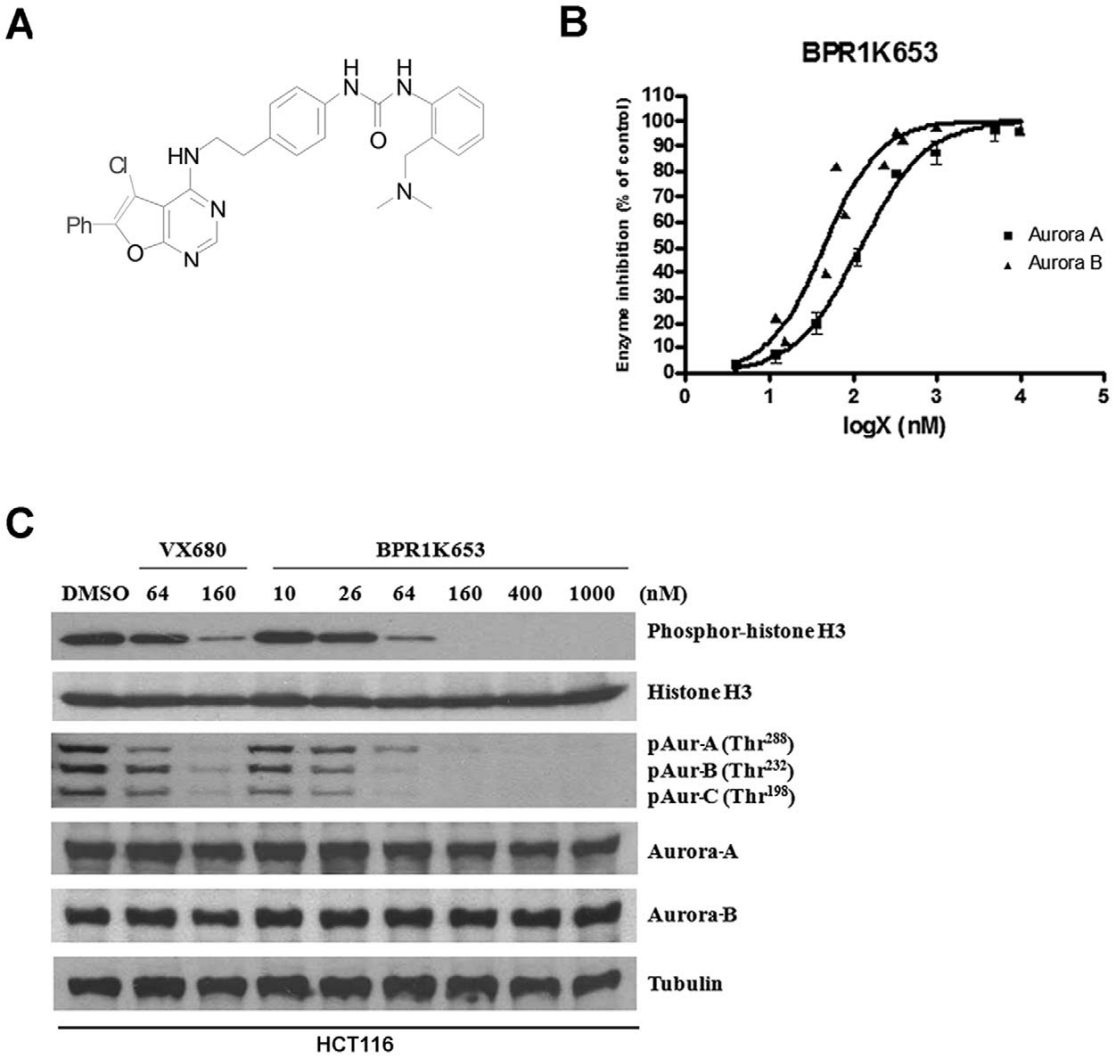
BPR1K653 selectively inhibits the activity of Aurora kinases *in vitro*. (A) Chemical structure of the anti-cancer compound BPR1K653. (B) BPR1K653 inhibited the activity of both Aurora-A and Aurora-B kinase as revealed by the *in vitro* kinase inhibition assay. (C) HCT116 cancer cells were treated with various concentrations of BPR1K653 and the commercially available pan-Aurora kinase inhibitor VX680, and the expression of various proteins were analyzed by Western blotting. Tubulin was used as the internal control.

*In vitro* kinase inhibition assay revealed that BPR1K653 (Figure 1A) inhibited the activity of Aurora-A and -B kinase with an IC_50_ value of 124 nM and 45 nM, respectively (Figure 1B and Table 1). The selectivity of BPR1K653 was then evaluated against different kinases. BPR1K653 exhibited less potency (i.e. IC_50_>10 µM) in inhibiting the activity of ALK, CHK1, cMET, EGFR, FLT3, VEGFR1 and VEGFR2 as compared to Aurora-A and Aurora-B kinase (Table 1). The cellular activity of BPR1K653 was also examined. Activation of Aurora-A kinase requires an auto-phosphorylation on the Thr288 residue, whereas phosphorylation of the Thr232 residue is an essential regulatory mechanism for Aurora-B activation [35], [36]. Here, Western blot analysis revealed that the amount of phosphor-Aurora-A, -B and -C kinase present in HCT116 cancer cells treated with a pan-Aurora kinase inhibitor, VX680 (positive control), was reduced in a concentration-dependent manner (Figure 1C). Reduction of phosphor-Histone H3 (Ser10), a direct substrate of Aurora-B kinase, is widely used as an indicator of Aurora kinase inhibition in cells. Here, VX680 also reduced the amount of phosphor-Histone H3 (Ser10) present in cells as expect (Figure 1C). Consistent with these findings, BPR1K653 induced a concentration-dependent decrease in phosphor-Aurora-A, -B and -C kinase in HCT116 cells. HCT116 cells treated with BPR1K653 also showed a concentration-dependent decrease in phosphor-Histone H3 (Figure 1C).

**Table 1:** BPR1K653 specifically inhibits Aurora-A and Aurora-B kinase.

Enzyme	Inhibition IC_50_ (nM)
***Aurora-A***	***124***
***Aurora-B***	***45***
ALK	>10000
CHK1	>10000
CHK2	2300
cMET	>10000
EGFR	>10000
FLT3	>10000
VEGFR1	>10000
VEGFR2	>10000

ALK, anaplastic lymphoma receptor tyrosine kinase; CL, total body clearance; CHK1, checkpoint kinase 1; CHK2 checkpoint kinase 2; cMET, c-Met tyrosine kinase; EGFR, epidermal growth factor receptor tyrosine kinase; FLT3, FMS-like tyrosine kinase; VEGFR1, vascular endothelial growth factor receptor 1 tyrosine kinase; VEGFR2, vascular endothelial growth factor receptor 2 tyrosine kinase.

### BPR1K653 inhibits the proliferation of multiple human cancer cell lines regardless of their tissue origins and p53 status

To determine whether BPR1K653 could inhibit cell proliferation, a panel of 11 different cancer cell lines was treated with BPR1K653. For comparison, cells were also treated with two well-characterized Aurora kinase inhibitors, VX680, and PHA739358. It has been demonstrated that loss of p53 function induces multidrug resistance in some types of cancer [37]. Here, results of the clonogenic assay revealed that BPR1K653 was effective (i.e. IC_50_<0.5 µM) against various types of cancer cells, including lung (A549), oral (HONE-1 and OECM-1) cervical (KB), colon (HT29), bladder (NTUB1) and leukemia/lymphoma (MV4-11 and IM9), regardless of their p53 status (Table 2). Moreover, the potency of BPR1K653 was shown to be higher than that of VX680 and PHA739358 in most of the tested cancer cell lines (Table 2). The IC_50_ values of VX680 and PHA739358 in various cancer cell lines (except in OECM-1 cells) were 2–10 folds higher than those of BPR1K653. The IC_50s_ of VX680 and BPR1K653 were equal in OECM-1 cells. Taken together, our results demonstrated that BPR1K653 is able to inhibit the proliferation of various types of cancer cell regardless of their tissue origins and p53 status.

**Table 2:** BPR1K653 exhibits anti-proliferative activity against various types of cancer cells.

				Aurora kinase inhibitors (nM)		
Cell line	tissue origin	p53 status	MDR1 status	BPR1K653	VX680	PHA-739358
**A549**	lung	wild-type	negative	9±0	111±9 (**12**)	56±8 (**6**)
**HT29**	colon	mutant	negative	12±2	160±33 (**15**)	48±8 (**4**)
**OECM-1**	oral	mutant	negative	135±10	123±37 (**1**)	642±68 (**5**)
**HONE-1**	oral	mutant	negative	11±0	20±2 (**2**)	59±16 (**5**)
**KB**	cervical	wild-type	negative	12±4	85±31 (**7**)	400±100 (**33**)
**NTUB1**	bladder	N/A	negative	8±4	73±6 (**9**)	405±134 (**51**)
**MV4-11**	leukemia	mutant	negative	5±0	15±4 (**3**)	86±11 (**17**)
**IM9**	lymphoma	wild-type	negative	4±2	31±16 (**8**)	450±12 (**113**)

Fold differences as compare to the IC50 of BPR1K653 are listed in brackets ( ).

### BPR1K653 is equally potent in inhibiting the growth of the multiple-drug resistance protein (MDR1) -expressing cancer cells

**Figure 2:**
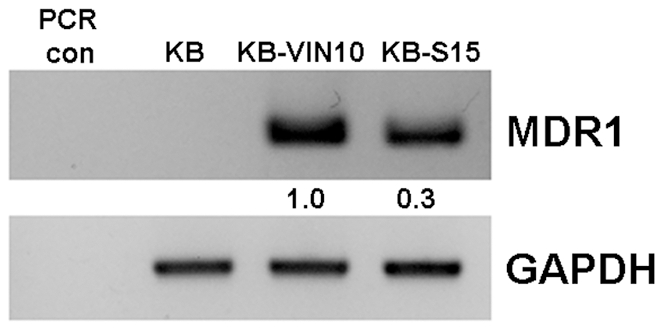
Level of MDR1 expression correlates to the level of resistance of VX680/PHA739358 in KB-VIN10 and KB-S15 cancer cells. Total mRNA was extracted from cells, and RT-PCR was performed to detect the expression of MDR1 in KB, KB-VIN10 and KB-S15 cells. GAPDH was used as internal control.

It has been widely demonstrated that over-expression of MDR1 (P-gp, drug efflux pump) induces drug resistance to various chemotherapeutic agents. To determine whether the potency of BPR1K653 is abrogated by MDR1 expression in cancer cells, three multidrug resistant MDR1-expressing cancer cell lines, KB-VIN10, KB-S15 and NTU0.017 [2], [38], [39], [40], were treated with BPR1K653. As shown in Table 3, the IC_50_ value of BPR1K653 to KB-VIN10 and KB-S15 was similar to those of the parental MDR1-negative KB cells. The IC_50_ of BPR1K653 to KB-VIN10, KB-S15 and KB were 14 nM, 11 nM and 12 nM, respectively. In addition, the IC_50_ value of BPR1K653 to the MDR1-expressing NTU0.017 cells was also similar to that of the parental MDR1-negative NTUB1 cells (Table 3). Previous studies revealed that Aurora kinase inhibitors, VX680 and PHA739358, are substrates of MDR1 [24], [25]. Consistently, all of our tested MDR1-expressing cancer cell lines showed cross-resistant to VX680 and PHA739358 (Table 3). In addition, the level of MDR1 expression correlated with the level of VX680/PHA-739358 resistance in KB-VIN10 and KB-S15 cancer cells (Figure 2). To further determine whether the potency of VX680 and PHA739358 in KB-VIN10, KB-S15 and NTU0.017 cells were actually affected by the expression of MDR1, cells were co-treated with the MDR1 modulator (negative regulator), verapamil, and cell viability was determined. Here, verapamil treatment (10 µM) was shown to be able to restore/enhance the sensitivity to both VX680 and PHA739358 in all of the tested MDR1-expressing cancer cells (Table 3). However, verapamil treatment could not further increase the sensitivity to BPR1K653 in both MDR-negative and MDR1-expressing cancer cells (data not shown). On the other hand, it has been demonstrated that a KB derived VP-16 resistant cancer cell line, KB-7D, over-expresses another type of the ATP-dependent multi-drug efflux protein, MPR1 [41]. Interestingly, the IC_50_ value of BPR1K653 to KB-7D was also similar to that of the parental MRP1-negative KB cells (Table 3).

**Table 3:** BPR1K653 exhibits anti-proliferative activity against various MDR1/MRP1-positive cancer cells.

			Treatments (nM)				
Cell line	Resistance	MDR1/MRP1 status	BPR1K653	VX680	VX680 + verapamil	PHA-739358	PHA-739358 + verapamil
**KB**	(parental)	negative	12±4	85±31	57	400±100	184
**KB-VIN10**	vincristine	**MDR1 ↑**	14±4 (**1**)	1400±140 (**16**)	60±0 (**1**)	>25,000 (**>63**)	1,400±200 (**8**)
**KB-S15**	paclitaxol	**MDR1 ↑**	11±4 (**1**)	272±20 (**3**)	46±8 (**1**)	4700 (**12**)	436 (**2**)
**KB-7D**	VP-16	**MRP1 ↑**	19 (**1.2**)	-	-	-	-
**NTUB1**	(parental)	negative	8±4	73±6	44	405±134	144
**NTU0.017**	paclitaxol	**MDR1 ↑**	10±4 (**1**)	6766±1078 (**93**)	121±24 (**3**)	>50,000 (**>123**)	1,380±700 (**10**)

Fold differences as compare to the IC50 in the respective parental cells are listed in brackets ( ).

### BPR1K653 induces endo-replication in both MDR1-negative and -positive cancer cells

**Figure 3:**
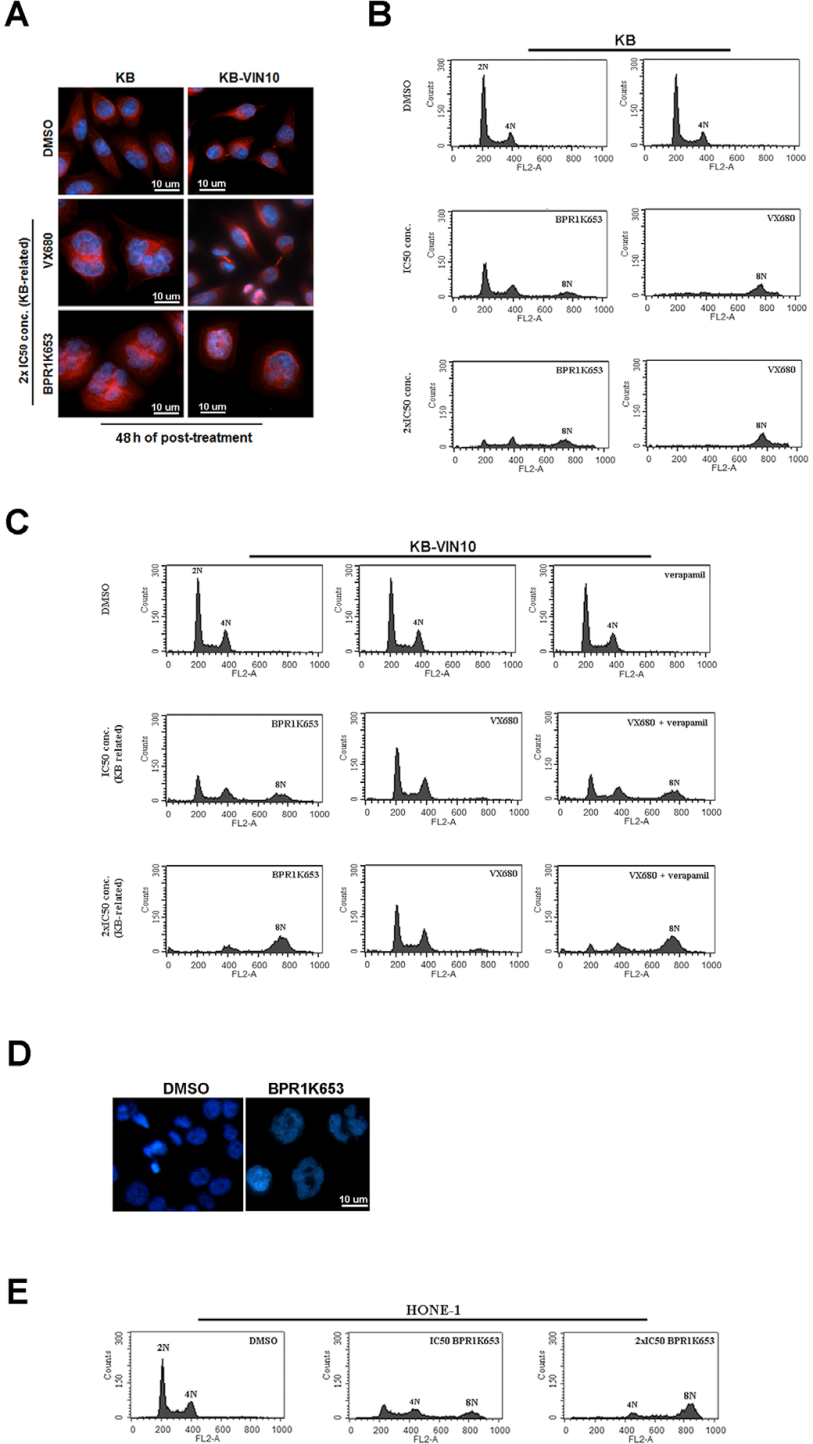
BPR1K653 induces endo-replication in both MDR1-negative and MDR1-expressing cancer cells. (A, B and C) KB and KB-VIN10 cells were treated with BPR1K653 and VX680 for 48 h. (A) Nucleus were stained blue with Hoechst dye and microtubules were labeled red with the Alexa Fluor®-tagged anti-tubulin antibody. (B and C) Cells were incubated with propidium iodide and subsequently analyzed by flow cytometry. (D and E) HONE-1 cells were treated with BPR1K653 for 48 h. (D) Nucleus were stained blue with the Hoechst dye. (E) Cells were incubated with propidium iodide and subsequently analyzed by flow cytometry.

Further experiments were performed to reconfirm the above findings that the effectiveness of BPR1K653 is not affected by the MDR1 expression in cells. Inhibition of Aurora kinases induces endo-reduplication of cells, indicating by the formation of polyploidy [14]. Here, results of immunofluorescence microscopy and flow cytometric analysis clearly showed that BPR1K653 induced the formation of polyploidy (populations >4N) in KB cells (Figure 3A and B, and Figure S1A). The MDR1-expressing KB-VIN10 cells treated with the same concentrations of BPR1K653 as had been applied to KB cells also induced the formation of polyploidy (Figure 3A and C, and Figure S1A). In contrast, VX680 only induced the formation of polyploidy in KB cells but not in KB-VIN10 cells under the same treatment concentrations (Figure 3A, B and C). However, formation of the polyploidy population was shown in KB-VIN10 cells co-treated with 10 µM of the MDR-inhibitor, verapamil, and VX680 (Figure 3C). These results are consistent with the findings of the above clonogenic assay that expression of MDR1 in cancer cells affects the effectiveness of VX680 but not of BPR1K653.

To determine whether BPR1K653 also induces endo-replication in cancer cell lines other than KB and its derivative, HONE-1 cells were treated with BPR1K653 and cellular contents were analyzed by microcopy and flow cytometry. Both immunofluorescence microscopy and flow cytometric analysis clearly showed that BPR1K653 promoted the formation of polyploidy (populations >4N) in HONE-1 cells in a concentration-dependent manner (Figure 3D and E).

### BPR1K653 reduces Histone H3 phosphorylation and cyclin B1 expression in both MDR1-negative and -positive cancer cells

**Figure 4:**
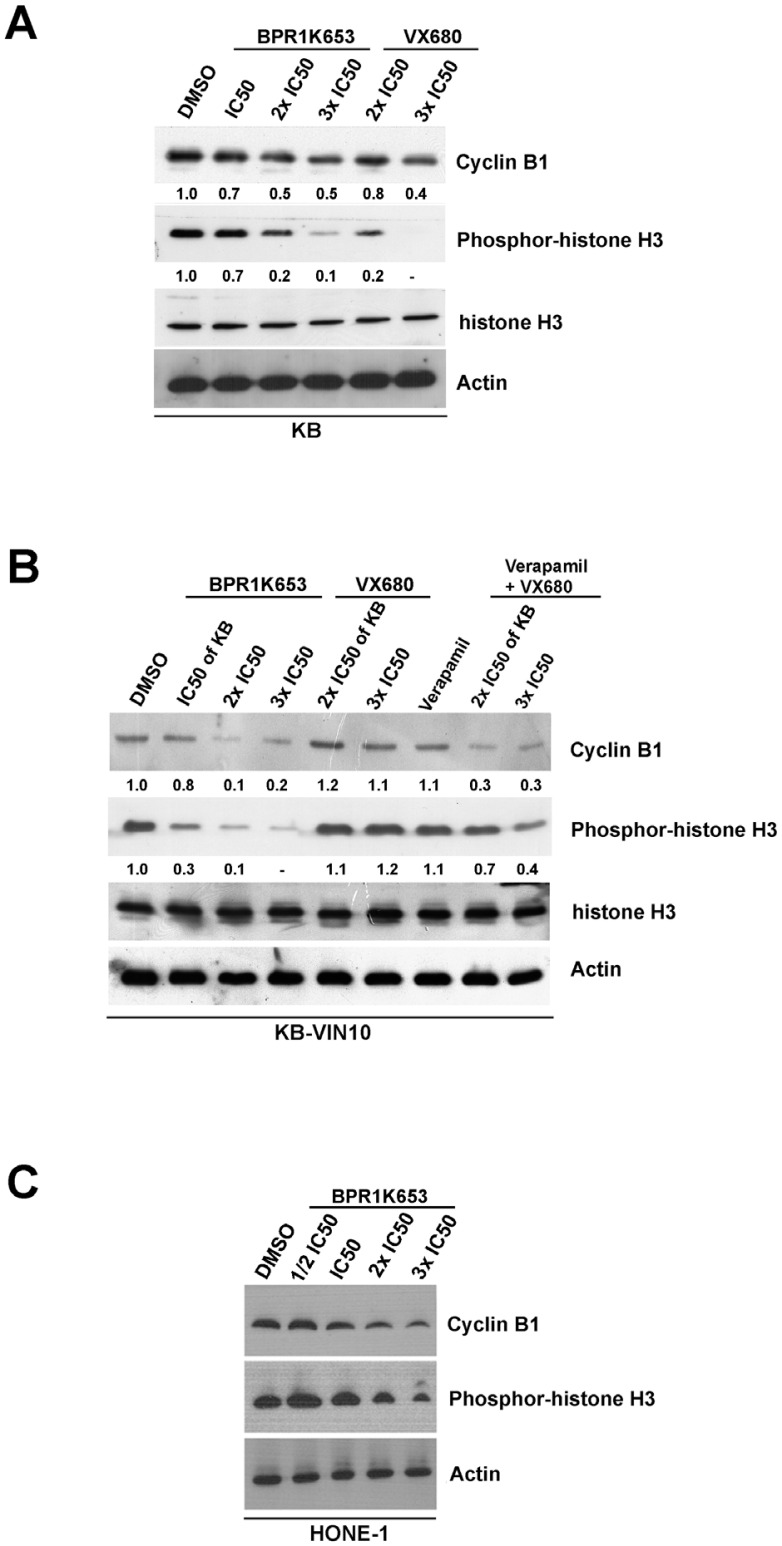
BPR1K653 down-regulates Histone H3 phosphorylation and cyclin B1 expression in both MDR1-negative and MDR1-expressing cancer cells. (A) KB cells were treated with BPR1K653 and VX680 for 48 h and expression of various proteins were determined by Western blot analysis. Relative band intensities were shown. (B) KB-VIN10 cells were treated with either BPR1K653 or VX680 with/without verapamil for 48 h, and expression of various proteins was determined by Western blot analysis. Relative band intensities were shown. (C) HONE-1 cells were treated with BPR1K653 for 48 h, and expression of various proteins was determined by Western blot analysis. Actin was used as the internal control.

Western blot analysis was performed to reconfirm that the effectiveness of BPR1K653 is not affected by the MDR1 expression in cancer cells. Histone H3 is a direct substrate of Aurora-B kinase, and endo-replicating cells usually show reduction of the expression of cyclin B1. In this experiment, inhibition of Histone H3 phosphorylation and down-regulation of cyclin B1 expression were shown in both KB and KB-VIN10 cells treated with the same concentrations, 12 (IC_50_), 24 (2× IC_50_) and 36 nM (3× IC_50_) of BPR1K653 in a concentration-dependent manner (Figure 4A and B). Consistent with these findings, VX680 treatment (i.e. 170 nM and 255 nM) also inhibited the phosphorylation of Histone H3 and the expression of cyclin B1 in KB cells (Figure 4A). However, same VX680 treatment could not induce the above molecular changes in the MDR1-expressing KB-VIN10 cells. Verapamil treatment (10 µM) was shown to restore the sensitivity to VX680 in KB-VIN10 cells, as indicated by a reduction in the Histone H3 phosphorylation and cyclin B1 expression (Figure 4B).

To determine whether BPR1K653 also reduces Histone H3 phosphorylation and cyclin B1 expression in cancer cell lines other than KB and its derivative, HONE-1 cells was treated with BPR1K653 and intracellular proteins were analyzed by Western blotting. Western blot analysis clearly demonstrated that both the phosphorylation of Histone H3 and expression of cyclin B1 were decreased in BPR1K653-treated HONE-1 cells (Figure 4C).

### BPR1K653 induces apoptosis in both MDR1-negative and -positive cancer cells

**Figure 5:**
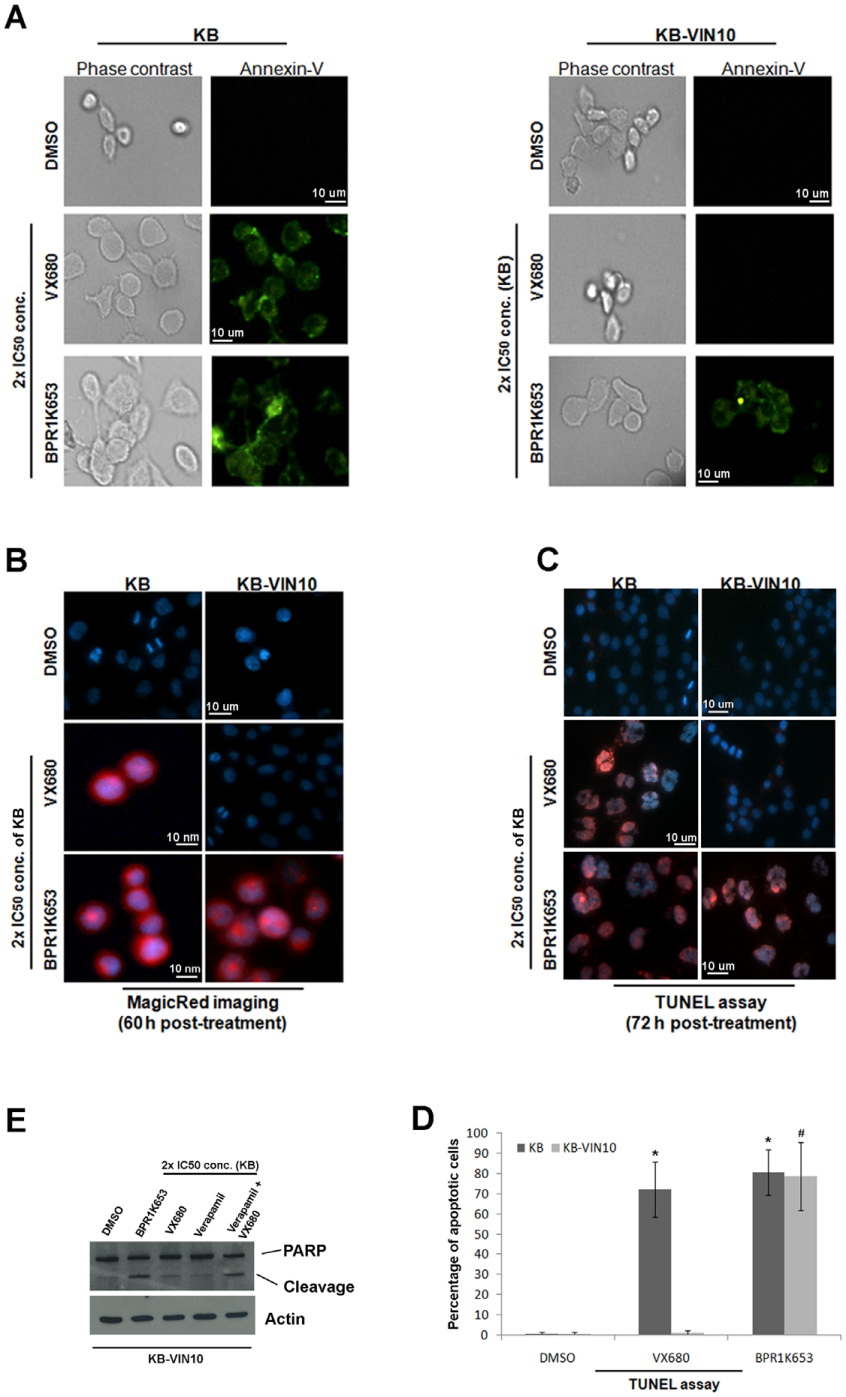
BPR1K653 induces apoptosis in both MDR1-negative and MDR1-expressing cancer cells. (A, B and C) KB and KB-VIN10 cells were seeded on 8-well chamber slides overnight. (A) Cells were treated with either BPR1K653 or VX680 for 48 h. Translocation of the phosphatidylserine molecule in cells was analyzed by Annexin-V-FLUOS assay and cells were viewed using an UV-enabled microscope. General cell morphology was visualized by phase-contrast microscopy. (B) Cells were treated with either BPR1K653 or VX680 for 60 h and MagicRed™-DEVD Real-time Caspase-3/-7 Activity kit (Immunochemistry Technologies LLC) was used to detect the activation of caspase-3/-7 in cells, as indicated by the red fluorescent emission. Nucleus was counter-stained blue by Hoechst 33342, and cells were viewed real-time using an UV-enabled inverted microscope. (C and D) Detection of cells with DNA fragmentation by TUNEL assay. KB and KB-VIN10 cells were treated with either BPR1K653 or VX680 for 72 h. DNA fragmentations were analyzed using the TMR-red *In Situ* Cell Death Detection kit. Nucleus with DNA fragmentation was stained red. Nucleus was counter-stained blue by DAPI. Cells were analyzed by an UV-enabled microscope. (C) Representative photos were shown. (D) Labeled cells were counted, and percentage of apoptotic cells was calculated as follows: Total amount of the red fluorescent labeled (DNA fragmented) nucleus available ÷ Total amount of the blue fluorescent labeled nucleus available×100. Experiments were repeated twice. (E) BPR1K653 induces the cleavage of PARP in KB-VIN10 cancer cells. KB-VIN10 cells were treated with either BPR1K653 (2× IC_50_ of KB) or VX680 (2× IC_50_ of KB) with/without verapamil for 72 h. The cleavage of PARP was determined by Western blot analysis. Actin was used as the internal control.

Previous studies revealed that targeting Aurora kinases induces cell endo-replication and subsequent cell apoptosis [14]. To determine whether BPR1K653 is able to induce apoptosis in both MDR1-positive and -negative cancer cells, KB and KB-VIN10 cells were treated with BPR1K653 and apoptotic properties were analyzed by Annexin-V, real-time caspase-3/-7 activity imaging and TUNEL assays. Here, both cytoplasmic volume and the size of nucleus were increased in the BPR1K653-treated KB and KB-VIN10 cells, indicating that BPR1K653 induced cell endo-replication as expected (Figure 5A and C, and Figure S1A). Translocation of the phosphatidylserine molecule from the inner-leaflet of cell membrane to the outer membrane indicates the occurrence of early apoptosis. Results of the Annexin-V assay showed that BPR1K653 induced the translocation of the phosphatidylserine molecule in both KB and KB-VIN10 cells, as indicating by the green fluorescent label (Figure 5A). BPR1K653 also induced the caspase-3/-7 activity and DNA fragmentation in both KB and KB-VIN10 cells under the same treatment conditions (Figure 5B, C, D, and Figure S1A). In contrast, VX680 only induced the translocation of the phosphatidylserine molecule, caspase-3/-7 activity and DNA fragmentation in KB cells and not in the MDR1-expressing KB-VIN10 cells (Figure 5A, B, C and D). Moreover, cleavage of PARP was only shown in the MDR1-expressing KB-VIN10 cells treated with either BPR1K653 or VX680/verapamil (co-treatment), and not with VX680 alone, as revealed by the Western blot analysis (Figure 5E).

BPR1K653 also induced apoptosis in HONE-1 cells, as indicated by the induction of caspae-3/-7 activity *in vitro* (Figure S1B).

### BPR1K653 suppresses the growth of both human MDR1-negative and -positive cancer xenografts *in vivo*

**Figure 6:**
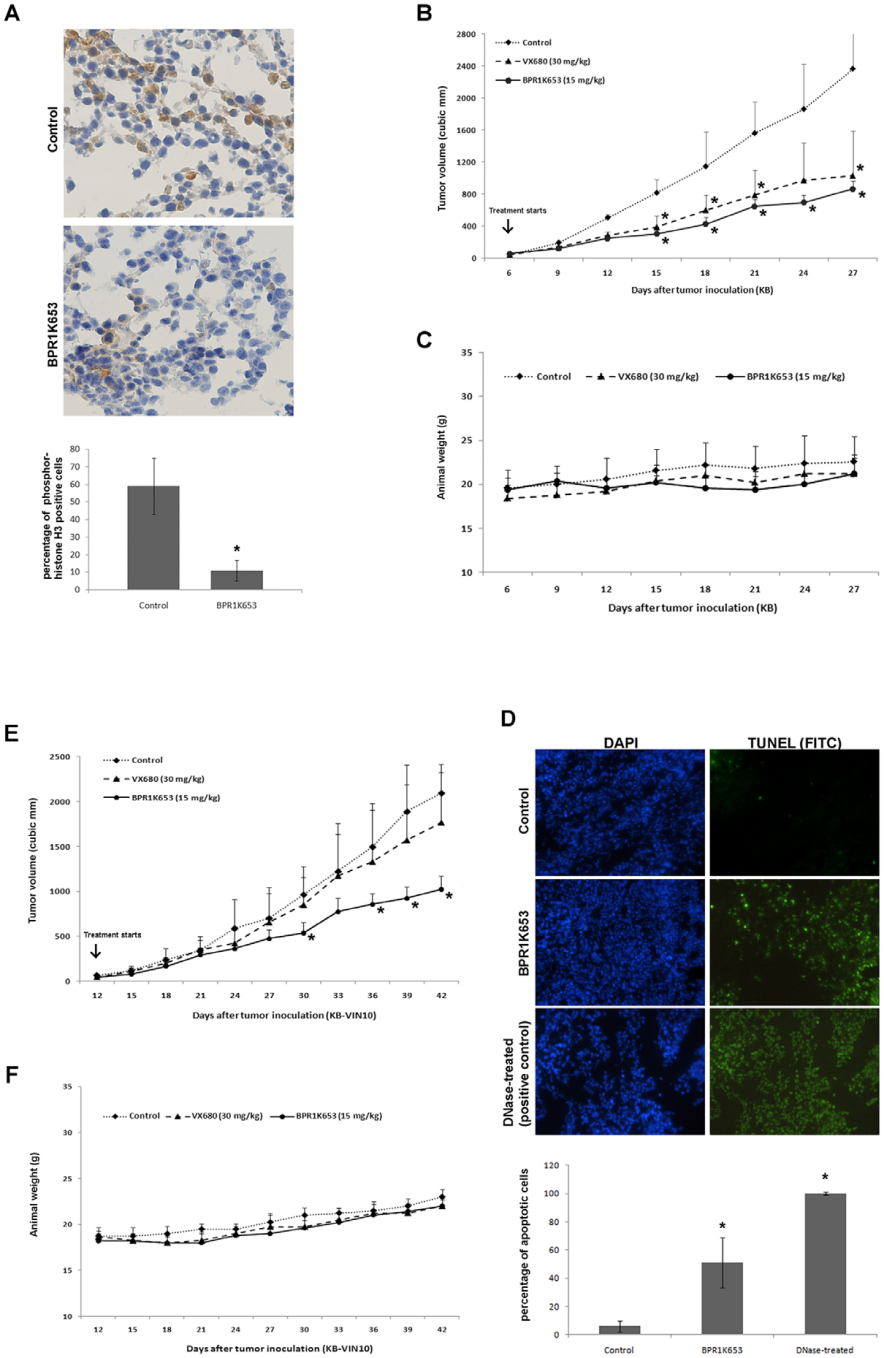
Inhibition of human xenografts growth *in vivo* by BPR1K653. (A, B, C and D) Nude mice bearing human cervical carcinoma KB xenografts were treated with vehicle control (⧫), 30 mg/kg VX680 for 5 days/week for 2 weeks (on days 6–10 and 13–17; ▴) or 15 mg/kg BPR0L075 for 5 days/week for 2 weeks (on days 6–10 and 13–17; •). (A) BPR1K653 treatment reduced the amount of the phosphor-Histone H3 positive cells present in tumor tissues. Immuno-histochemical analysis of the expression of phosphor-Histone H3 in the tumor tissue sections 24 h after the second BPR1K653 administration. Nucleus was stained blue/purple by hematoxylin and phosphor-Histone H3 was labeled in brown colour. Labeled cells were counted, and percentage of the phosphor-Histone H3 positive cells present in tumor tissues was calculated as follows: Total amount of cells with brown color labeled ÷ Total amount of cells available×100. Experiment was repeated twice. A statistically significant difference in the amount of phosphor-Histone H3 positive cells present in tumor tissues in mice treated with control versus BPR1K653 is denoted by “*”. *p<0.05. (B) Measurement of tumor volume. A statistically significant difference in tumor size in mice treated with control versus BPR1K653 and VX680 is denoted by “*”. *p<0.05. (C) Measurement of animal weight. (D) TUNEL analysis of the tumor tissue sections 12 days post-BPR1K653 treatment. Tumor tissue sections were analyzed by the FITC *In Situ* Cell death detection kit and fluorescent microscopy. Tissue treated with DNase was used as the positive control. Green fluorescence labeled nucleus indicates the induction of DNA fragmentation. Experiment was repeated twice. Quantitative analysis was shown. A statistically significant difference in the amount of apoptotic cells present in tumor tissues in mice treated with control versus BPR1K653 is denoted by “*”. *p<0.05. (E and F) Nude mice bearing the P-gp170/MDR-expressing KB-VIN10 xenograft was treated with vehicle control (⧫), 30 mg/kg VX680 for 5 days/week for 3 weeks (on days 12–16, 19–23 and 26–30; ▴) or 15 mg/kg BPR0L075 for 5 days/week for 3 weeks (on days 12–16, 19–23 and 26–30; •). (E) Measurement of tumor volume. A statistically significant difference in tumor size in mice treated with control versus BPR1K653 and VX680 is denoted by “*”. *p<0.05. (F) Measurement of animal weight. Data are the mean ± SD of tumor volume (mm^3^) at each time point (n = 5; *P<0.05).

Although the above results showed that BPR1K653 exhibits potent anti-cancer effect *in vitro*, experiments were performed to determine whether BPR1K653 is also able to inhibit the activity of Aurora kinases and the growth of both MDR1-negative/positive tumors *in vivo*. KB cells were grown as *s.c.* tumors in nude mice. When well-established KB xenografts were palpable with tumor size of ∼75 mm^3^, mice were randomized into vehicle control and treatment groups of five animals each. The treated mice received either 15 mg/kg of BPR1K653 or 30 mg/kg of VX680 *i.p.* for 5 days/week for 2 consecutive weeks. Results of the immuno-histochemical analysis of the tumor tissue sections showed that administration of BPR1K653 reduced the amount of phosphor-Histone H3 positive cells present in tumor tissues as compared to the control (10% vs 60%) (Figure 6A). A decrease in the rate of tumor growth in mice treated with either BPR1K653 or VX680 5 days/week for 2 consecutive weeks was also observed. There was a ∼73% decrease in tumor volume on day 30 in the animals treated with BPR1K653 (P<0.05). In addition, there was a ∼68% decrease in tumor volume on Day 30 in the animals treated with VX680 (P<0.05; Figure 6B). BPR1K653 was well-tolerated at the dosage of 15 mg/kg with no signs of toxicity in the KB xenograft tumor model as the loss of body weight after treatment was less than 10% in the treatment group as compare to the control group (Figure 6C). To determine whether the inhibition of tumor growth in BPR1K653-treated mice was related to the increases of apoptotic cancer cell populations, tumors were surgically removed from the mice 12 days post-treatment and tissue sections were analyzed by TUNEL assay. Results of the TUNEL assay showed that the amount of apoptotic cells present in the tumor tissue of BPR1K653-treated mice was significantly higher than those in the control mice (55% vs 7%) (Figure 6D). This is consistent with the result of the above *in vitro* experiment that BPR1K652 is able to induce cancer cells apoptosis.

Notably, BPR1K653 is also as effective toward MDR1-expressing tumor xenograft as it is in cultured MDR1-expressing cells. Here, KB-VIN10 tumor xenograft was used to evaluate the efficacy of BPR1K653 against MDR1-expressing tumor *in vivo*. Due to the slow growing properties of KB-VIN10, the treated mice received either 15 mg/kg of BPR1K653 or 30 mg/kg of VX680 *i.p.* for 5 days/week for 3 consecutive weeks instead of 2 weeks as in KB-implanted mice. In comparison to the control mice, growth of KB-VIN10 tumor was significantly inhibited in mice treated with 15 mg/kg of BPR1K653. There was a ∼50% decrease in tumor volume on Day 42 in the animals treated with BPR1K653 (P<0.05). In contrast, VX680 did not exhibit significant tumor growth inhibitory effect in mice transplanted with KB-VIN10 cells (Figure 6E). Moreover, BPR1K653 was well-tolerated at the dosage of 15 mg/kg (5 days/week for 3 consecutive weeks) with no signs of toxicity in the KB-VIN10 xenograft tumor model as the loss of body weight after treatment was less than 10% in the treatment group as compare to the control group (Figure 6F). Thus, BPR1K653 exerts potent anti-tumoral efficacy toward both MDR-negative and MDR-expressing tumor xenografts.

### Pharmacokinetics of BPR1K653 in rats

Finally, pharmacokinetic studies of BPR1K653 were accessed over a 24 h period to examine plasma concentrations of BPR1K653 after a single intravenous administration (Table 4). After a single administration of BPR1K653 at a dosage of 5 mg/kg to rats, BPR1K653 achieved a maximum plasma concentration of 10 µM (5463 ng/mL) at 2 min after dosing, and the estimated BPR1K653 plasma concentration remained at a concentration of 3.9 nM (2.1 ng/mL) 24 h after dosing. The plasma half-life, total body clearance, and volume of distribution at the steady state (Vss) were 3.9±0.7 h, 49.3±10.6 mL/min/kg and 10.6±5.1 L/kg, respectively.

**Table 4:** Pharmacokinetic proflile of the Aurora kinase inhibitor, BPR1K653.

**Plasma half life (t_1/2_)**	3.9 hours
**Total body clearance (CL)**	49.3 mL/min/kg
**Volume of distribution at the steady state (V_ss_)**	10.6 (ng/kg)
**Area under the curve (AUC_(0-inf)_)**	1752 ng/mL*h

*In rats (dosage of BPR1K653 - 5 mg/kg, *i.v.*).

## Discussion

Aurora kinases have emerged as key regulators of mitosis and evidence indicates abnormalities in their expression and activity are closely related to the development and progression of various cancers. In this study, we have developed a novel pan-Aurora kinase inhibitor BPR1K653 and further demonstrated its efficacy in targeting various types of cancers *in vitro*. Our pervious x-ray co-crystallography studies had demonstrated the physical interactions between the precursor compound of BPR1K653 and Aurora kinases [42], and the current *in vitro* kinase inhibition study has confirmed the target specificity of BPR1K653. Consistent with the molecular changes observed in cells treated with Aurora-B kinase specific siRNA oligos and with different pan-Aurora kinase inhibitors such as VX680 and SNS-314 [14], [43], [44], BPR1K653 treatment also induces endo-replication of cells and reduces amount of phosphorylated Histone H3 present in cells. In addition, BPR1K653 is able to induce cancer cell apoptosis but not autophagy (Figure S2), which is the common result in cells treated with Aurora kinase inhibitors [43]. Interestingly, BPR1K653 is active in all of the tested p53-wildtype/-negative/-mutant cancer cell lines at low nano-molar concentrations (IC_50_<20 nM), despite limited ability of another pan-Aurora kinase inhibitor VX680 to induce endo-replication and subsequent apoptosis has been shown in cancer cells with normal p53-dependent post-mitotic checkpoint function in other study [14]. Taken together, BPR1K653 is selectively inhibiting Aurora kinases, and unlike VX680, it is able to target various types of cancer cells regardless of their p53 status.

Drug resistance is a common problem in the management of neoplastic diseases, and the effectiveness of many chemotherapeutic drugs is limited by the fact that they are substrates for the efflux pump MDR1 (P-gp170). For example, the Aurora kinase inhibitor AZD1152/AZD1152-HQPA (Barasertib) was shown to be the substrate of MDR1 [24]. Moreover, our reference Aurora kinase inhibitors, VX680 (Tozasertib) and PHA-739358 (Danusertib), were previously shown ineffective in targeting the MDR1-expressing SA-Dx5 (doxorubicin resistant), MB-231-PTX and H460-PTX (both paclitaxel resistant) cancer cells by other investigators [25]. In this study, BPR1K653 was shown to be equally effective against two KB-derived MDR1-positive cancer cell lines (KB-VIN10 and KB-S15) and one NTUB1-dervided MDR1-positive cancer cell line (NTU0.017) *in vitro*. This feature is distinct from those of the well-characterized Aurora kinase inhibitors, VX680 and PHA-739358, because our tested MDR1-positive cancer cells are more resistant to these chemotherapeutic agents than their parental MDR1-negative cells. Indeed, co-incubation of the MDR1 inhibitor, verapamil, was shown to be effective in re-sensitizing the MDR1-expressing cancer cells to both VX680 and PHA-739358, whereas the same treatment could not enhance the sensitivity to BPR1K653 in neither MDR1-negative (KB and NTUB1) nor MDR1-expressing cells (KB-VIN10, KB-S15 and NTU0.017). Importantly, BPR1K653 is also effective in inhibiting the growth of both MDR1-negative KB and MDR1-expressing KB-VIN10 cancer cells *in vivo*, further supporting the hypothesis that over-expression of the common drug efflux pump MDR1 could not interfere with the efficacy of BPR1K653 in targeting cancer cells. Since chemotherapeutic compounds such as paclitaxel, vincristine (anti-microtubule agents), doxorubicin (DNA intercalating agent), tretinoin (*all-trans* retinoic acid), mitoxantrone, VP-16 (topoisomerase II inhibitors) and imatinib (tyrosine kinase inhibitor) are all substrates of the drug efflux pump MDR1, the use of BPR1K653 may be beneficial in patients that are resistant to the above compounds after prolonged therapeutic treatments [30], [31], [32].

It has been known that most newly-developed anti-cancer compounds that perform well *in vitro*, do not progress to the clinical stage due various factors such as unfavorable pharmacokinetic properties and reduced potency *in vivo*. In this study, we have shown that BPR1K653 exhibits favorable pharmacokinetic properties *in vivo*. The maximum achievable plasma concentration of BPR1K653 (10 µM, 5463 ng/mL) after a single administration at a dosage of 5 mg/kg to rat is more than 80-fold and 200-fold above the *in vitro* kinase inhibition IC_50_ of Aurora-A and -B kinase respectively. Even though at 24 h after dosing, the plasma levels of BPR1K653 (2 ng/mL) was still high enough to inhibit the activity of both Aurora-A and Aurora-B kinase. In addition, the high Volume of distribution at the steady state (Vss) value (10.6 l/kg) indicates that the distribution of BPR1K653 into deep compartments, including tumor and tissues is expected. Taken together, these favorable pharmacokinetic properties suggest that BPR1K653 dosing once a day is sufficient for continuous inhibition of the activity of both Aurora-A and Aurora-B kinase.

In conclusion, BPR1K653 is a potent pan-Aurora kinase inhibitor that is able to target cancer cells regardless of their tissue origins, MDR1 or p53 status. These key features distinguish this compound from other previously developed Aurora kinase inhibitors and anti-cancer compounds. At the molecular level, results of this study suggest that BPR1K653 can be used as a tool to study the molecular functions of Aurora kinases in the MDR1-induced drug resistant cancer cells in the future. As BPR1K653 exhibits favorable pharmacokinetic properties in animal models, further evaluations are warranted to determine whether BPR1K653 is also effective in clinical situations.

## Materials and Methods

### Ethics statement

The animals used in this study were housed and the experiments were carried out at an International Association for Assessment and Accreditation of Laboratory Animal Care-accredited animal facility at the National Health Research Institutes, Tainan, Taiwan R.O.C.. The Institutional Animal Care and Use Committees for Biotechnology and the National Health Research Institutes approved uses of animals in these studies (approval number: NHRI-IACUC-099070).

### The Aurora-kinase inhibitor BPR1K653

Our previous structure-activity relationship studies (SAR) and X-ray co-crystallographic analysis had indentifed novel furanopyrimidine as Aurora kinase inhibitor [42]. The pan-Aurora kinase inhibitor BPR1K653 (Figure 1A) was synthesized from 4-chloro-6-phenylfuro[2,3-d]pyrimidine, which was originally obtained via a well-established 3-step process [42].

### Cell culture

Human cervical carcinoma KB cells (this cell line was originally believed to be derived from an epidermal carcinoma of the mouth but has now been shown to have HeLa characteristics, purchased from ATCC®), nasopharyngeal carcinoma HONE-1 cells [45], colorectal carcinoma HT29 cells (purchased from ATCC®), oral squamous cell carcinoma OECM-1 cells [46], leukemia MV4-11 cells (purchased from ATCC®), myeloma IM9 cells [47] were maintained in RPMI 1640 medium supplied with 5% fetal bovine serum. Human lung adenocarcinoma A549 cells and NTUB1 bladder cancer cells were maintained in RPMI supplied with 10% fetal bovine serum. KB-derived MDR1-expressing cell lines (*i.e.* KB-VIN10 and KB-S15) and NTUB1-dervided MDR1-expressing cell line (*i.e.* NTU0.017) were maintained in growth medium supplemented with 10 nM vincristine, 15 nM and 17 nM paclitaxel respectively. KB-VIN10 cells were generated in pervious study by vincristine selection and displayed over-expression of P-gp170/MDR1 [40], [48], [49]. KB-S15 and NTU0.017 cells were generated in previous studies by paclitaxel selection and also displayed over-expression of P-gp170/MDR1 [40], [50], [51]. KB-derived MRP1-expressing cell line, KB-7D, was maintained in growth medium supplemented with 7 µM VP-16. KB-7D cells were generated in pervious study by VP-16 selection and displayed over-expression of MRP1 [41].

### Kinase inhibition assay

Aurora-A and Aurora-B kinase - The recombinant GST-tagged Aurora-A (residues S123-S401) containing kinase domain was expressed in Sf9 insect cells. The recombinant full length His-tagged Aurora-B (residues M1∼A344) was purchased from Invitrogen (PV3970). The kinase assay were carried out in 96-well plates with the tested compound at either 37°C (Aurora-A) for 90 min or 30°C (Aurora-B) for 120 min.

ALK – The recombinant His-tagged ALK (residues V1058-P1620) containing kinase domain was expressed in Sf9 insect cells. The kinase assay was carried out in 96-well plates with the tested compound at 30°C for 120 min.

CHK1/2 – The recombinant His-tagged CHK1 (residues M1-T476) or CHK2 (residues M1-L543) containing kinase domain were expressed in Sf9 insect cells. The kinase assay was carried out in 96-well plates with the tested compound at 30°C for 120 min.

c-Met – The recombinant GST-tagged c-Met (residues K956-S1390) containing kinase domain was expressed in Sf9 insect cells. The kinase assay was carried out in 96-well plates with the tested compound at 30°C for 120 min.

EGFR – The recombinant GST-tagged EGFR (residues G696-G1022) containing kinase domain was expressed in Sf9 insect cells. The kinase assay was carried out in 96-well plates with the tested compound at 37°C for 60 min.

FLT3 – GST-tagged FLT3-KDWT containing the FLT3 kinase catalytic domain (residues Y567∼S993) were expressed in Sf9 insect cells. The FLT3WT Kinase-Glo assays were carried out in 96-well plates at 30°C for 4 h with the tested compound.

VEGFR1/2 – The recombinant GST-tagged VEGFR1 (residues R781-I1338) or VEGFR2 (residues V789-V1356) containing kinase domain were expressed in Sf9 insect cells. The kinase assay was carried out in 96-well plates with the tested compound at 30°C for 120 min.

Composition of the reaction buffers used in different kinase inhibitory assays is described in Figure S3.

### Clonogenic assay

Two hundred cells in logarithmic growth phase were seeded in a 6-well plate. The cells were exposed to various concentrations of the test drugs for a three-generation period. At the end of the incubation period, cells were fixed and stained with 50% ethanol containing 0.5% methylene blue for 30 min. The plates were washed five times with water and allowed to air-dry. Colonies were countered manually. The IC_50_ value resulting from 50% inhibition of cell growth was calculated graphically as a comparison with the growth of the control group. Each value represents the average of at least three independent experiments run in triplicates.

### Cell cycle analysis

Cell cycle progression was monitored using flow cytometry. After drug treatment, cells were trypsinized, washed with PBS and fixed in 80% ethanol at −20°C for 1 h. The fixed cells were stained with propidium iodide (containing RNase) at room temperature in the dark for 20 min. The DNA content was determined by a fluorescence-activated cell sorting IV flow cytometer (BD Biosciences, Franklin Lakes, NJ). For each analysis, 10,000 cells were counted and the percentage of cells in each phase was calculated using the ModFit LT software (Verity Software House, Topsham, ME). Experiments were repeated independently at least three times.

### RT-PCR of MDR1

Total RNA was extracted with using TRIzol reagent (Invitrogen, Carlsbad, CA) and complementary DNA was synthesized from RNA with the SuperScript™ First-Strand Synthesis System (Invitrogen, Carlsbad, CA). Polymerase chain reaction was performed with target-specific primers. MDR1 sense (forward) primer: 5′-GCCTGGCAGCTGGAAGACAAATRCACAAAATT-3′; MDR1 anti-sense (reverse) primer: 5′-CAGACAGCAGCTGACAGTCCRAGAACAGGACT-3′; GAPDH sense (forward) primer: 5′-ACCACAGTCCATGCCATCAC-3′ and GAPDH anti-sense (reverse) primer: 5′ TCCACCACCCTGTTGCTGTA-3′.

### SDS-PAGE and Western Blot Analysis

Cells were lysed with ice-cold lysis buffer (10 mM Tris, 1 mM EDTA, 1 mM DTT, 60 mM KCl, 0.5% NP-40 and protease inhibitors). Total cell lysates were resolved on 10% and 12% polyacrylamide SDS gels under reducing conditions. The resolved proteins were electrophoretically transferred to PVDF membranes (Amersham Life Science, Amersham, U.K.) for Western blot analysis. The membranes were blocked with 5% non-fat milk at room temperature for two hours, washed twice with TBST (1% Tween) and then incubated with either anti-phosphorylated Aurora-A/-B/-C kinase antibody (#2914S, Cell Signaling Technology, Danvers, MA), anti-Aurora-A and -B kinase antibody (#ab1287 and #ab2254, Abcam, Cambridge, MA), anti-phosphorylated Histone H3 antibody (#9701, Cell Signaling Technology, Danvers, MA), anti-Histone H3 antibody (#9715, Cell Signaling Technology, Danvers, MA), anti-Cyclin B1 antibody or anti-Actin antibody (#sc-245 and #sc-130065, Santa Cruz Biotechnology, Santa Cruz, CA) overnight at 4°C. Membranes were washed twice with TBST then subsequently incubated with a horseradish peroxidase-conjugated secondary antibody (Santa Cruz Biotechnology, Santa Cruz, CA) for 1 hour at room temperature. Immunoreactivity was detected by Enhanced Chemiluminescence (Amersham International, Buckingham, U.K.) and autoradiography. Experiments were repeated independently at least two times.

### Annexin V assay

Cells were cultured in chamber-slides, incubated with test agents for 48 h, and washed twice with PBS. Cells were labeled with Annexin-V-FLUOS reagent (catalog number #11858777001, Roche, Indianapolis, IN) for 30 min at room temperature. The cells were analyzed by fluorescence microscopy.

### Real-time Caspase-3/-7 activity imaging

Caspase-3/-7 activity was analyzed with the MagicRed™ DEVD real-time caspase activity detection kit (catalog number #935, Immunochemistry Technologies LLC, Bloomington, MN). Briefly, cells were cultured in chamber-slides and incubated with test agents for various durations. Cells were then incubated with the Caspase-3/-7 substrate MR-(DEVD2) in culture medium for 1 hour, and then co-incubated with Hoechst 33342 for 15 min. Cells were viewed with a UV-enabled inverted-microscope at an excitation wavelength of 540 nm–560 nm and emission at 610 nm. Experiments were repeated independently at least two times.

### Visualization of apoptosis by the TUNEL assay

Under *in vitro* conditions, cells were seeded and cultured in 8-well chamber-slides, and treated with various compounds. The treated cells were washed with PBS, fixed with 4% paraformaldehyde for 30 min on ice, and permeabilized with PBST at room temperature. Apoptotic cells were stained by the TUNEL agent using the TMR (red) *In-Situ* Apoptosis Detection Kit (catalog number #12156792910, Roche Diagnostic, Mannheim, Germany). Cells were counterstained with DAPI to detect the nucleus, and examined by fluorescence microscopy. Amount of red fluorescence labeled (DNA fragmented) cells were counted and percentage of apoptotic cells were calculated as follows: Amount of the red fluorescence labeled cells ÷ Total cells available×100. Experiments were repeated independently at least two times.

Under *in vivo* conditions, tumors were dissected from the euthanized mice and instantly stored under −80°C. Tumor tissue sections were prepared from the use of cryostats (Leica Microsystems, Buffalo Grove, IL), and subsequently fixed with ice-cold methanol. Tissue sections were stained by the TUNEL reagent using Fluorescent (green) *In-Situ* Apoptosis Detection Kit (catalog number #11684795910, Roche Diagnostic, Mannheim, Germany). Cells were counterstained with DAPI to detect nucleus, and examined by fluorescence microscopy. Amount of green fluorescence labeled (DNA fragmented) cells were counted and percentage of apoptotic cells were calculated as follows: Amount of green fluorescence labeled cells ÷ Total cells available×100. Experiments were repeated independently at least two times.

### Animals and implantation of cancer cells

Male nude mice (5–6-weeks-old) were purchased from the National Laboratory Animal Centre (Taiwan R.O.C.). The animals were *s.c.* implanted with 5×10^5^ KB cells or 1×10^6^ KB-VIN10 cells mixed with equal volume of Matrigel (Becton Dickinson) in 0.1 mL at one flank per mouse via a 22-gauge needle. Tumor growth was examined twice a week after implantation, and the volume of tumor mass was measured with an electronic caliper and calculated as 1/2×length×width^2^ in mm^3^.

### Drug treatments and monitoring of the *in vivo* anti-tumor activity

BPR1K653 was dissolved completely in a vehicle mixture of DMSO/cremophor/saline (1∶2∶7). Selected dose of BPR1K653 was decided base on the following conditions: 1/2 of the dosage that caused noticeable body weight loss (>10%) in the treated mice during toxicity study. In the KB xenograft study, when the size of a growing tumor reached ≥75 mm^3^, the xenograft tumor-bearing nude mice were treated with either BPR1K653 or VX680 *i.p.* (5 mice per treatment group) at a dosage of 15 mg/kg or 30 mg/kg, respectively, for 5 days/week for 2 consecutive weeks. In KB-derived MDR1-overexpressing KB-VIN10 xenograft study, mice were treated with either BPR1K653 or VX680 (5 mice per treatment group) at a dosage of 15 mg/kg or 30 mg/kg respectively for 5 days/week for 3 consecutive weeks. The control group (5 mice) was treated with vehicle mixture only. Tumor size and animal body weight were measured every three days after drug treatment. Toxicity was evaluated based on the body weight reduction. At the end of the experiments (tumor size of the control >2000 mm^3^), animals were euthanized with carbon dioxide.

### Immunohistochemistry

Tumors were harvested and instantly stored at −80°C. Frozen cryostat sections were fixed with ice-cold methanol for 10 min. After washing with PBS, endogenous peroxidase was blocked using 3% hydrogen peroxide in TBS for 5 min. Immunostaining process was carried out according to the user's manual of the ABC Peroxidase Staining Kit (Pierce Biotechnology, Rockford, IL). Briefly, the tissues were incubated with a protein-blocking solution for 20 min, and subsequently stained with an anti-phosphorylated Histone H3 (Ser10) polyclonal antibody for 1 hour at room temperature. Then, the samples were incubated with the ABC reagent for 30 min, and subsequently incubated with the metal enhanced DAB substrate. The sections were counterstained with hematoxylin.

### Pharmacokinetic studies of BPR1K653 in rats

Male Sprague-Dawley rats weighing 300–400 g each (8–12 weeks old) were obtained from BioLASCO, Taiwan Co., Ltd., Ilan, Taiwan. Animals were surgically prepared with a jugular-vein cannula one day prior to dosing and fasted overnight (∼18–20 h) prior to dosing. Water was available ad libitum throughout the experiment. Single 5 mg/kg dose of BPR1K653, as a DMA/PEG (20/80, v/v) solution, was separately administered to groups of 3 rats each intravenously by a bolus injection via the jugular-vein cannula. At 0 (prior to dosing), 2, 5, 15 and 30 min, and at 1, 2, 4, 6, 8 and 24 h after dosing, a blood sample (0.15 mL) was collected from each animal via the jugular-vein cannula and stored in ice (0–4°C). Plasma was separated from the blood by centrifugation (14,000 g for 15 min at 4°C in a Beckman Model Allegra™ 6R centrifuge) and stored in a freezer (−20°C). All samples were analyzed for the parent drug by LC-MS/MS. LC/MS/MS conditions: The chromatographic system consisted of an Agilent 1200 series LC system and an Agilent ZORBAX Eclipse XDB-C8 column (5 µm, 3.0×150 mm) was connected to a MDS Sciex API3000 tandem mass spectrometer, which was equipped with a Turbo V™ ESI in the positive scanning mode at 600°C. Data was acquired via the multiple reactions monitoring (MRM) system. The MS/MS ion transitions were monitored at m/z of 541.4/106.4 for BPR1K653. The collision energy of 58.0 V was used for the analyst, BPR1K653. A gradient HPLC method was employed for the separation. Mobile phase A consisted of water containing 0.1% formic acid, and mobile phase B consisted of acetonitrile. The gradient profile was shown as follows (min/%B): 0.0–1.2/5, 1.3–3.9/95, 4.0–5.0/5. The flow rate was set to be 1.5 mL/min. The auto-sampler was programmed to inject 15 µL sample aliquots in every 5 min. The retention time of BPR1K653 was 2.39 min. Plasma concentration data were analyzed with non-compartmental method.

### Statistical analysis

For all statistical analysis, values were expressed as mean ± SD. Values were compared using Student's *t*-test. P<0.05 was considered significant.

## Supporting Information

Figure S1 **BPR1K653 induces cell endo-replication and apoptosis.** (A) BPR1K653 induces endo-replication and subsequent DNA fragmentation in both KB and KB-VIN10 cells. Cells were treated with either DMSO or BPR1K653 for various durations, and nucleus was stained with Hoechst 33342. (B) BRP1K653 induces caspase-3/-7 activity in HONE-1 cancer cells. Cells were treated with either BPR1K653 for 60 h and MagicRed™-DEVD Real-time Caspase-3/-7 Activity kit (Immunochemistry Technologies LLC) was used to detect the activation of caspase-3/-7 in cells, as indicated by the red fluorescent emission. Nucleus was counter-stained blue by Hoechst 33342, and cells were viewed real-time using an UV-enabled inverted microscope. General cell morphology was visualized by phase-contrast microscopy.
(TIF)

Figure S2 **BPR1K653 did not interfere with the process of autophagy in cancer cells.** KB cells were treated with either DMSO (negative control) or BPR1K653 (48 h or 72 h) under full serum conditions. Cells cultured drug-free under reduced serum conditions were used as a positive control. Expression of various proteins was determined by Western blotting. The level of conversion of LC3-I to LC3-II provides an indicator of autophagic activity.
(TIF)

Figure S3 **Details of the composition of the reaction buffers used in different kinase inhibition assay.**
(DOC)
