# Supplementary figures and images for: BPR1K653, a Novel Aurora Kinase Inhibitor, Exhibits Potent Anti-Proliferative Activity in MDR1 (P-gp170)-Mediated Multidrug-Resistant Cancer Cells
Source: PLoS One. 2011 Aug 24;6(8):e23485. doi: 10.1371/journal.pone.0023485 (PMC3160846; doi:10.1371/journal.pone.0023485)

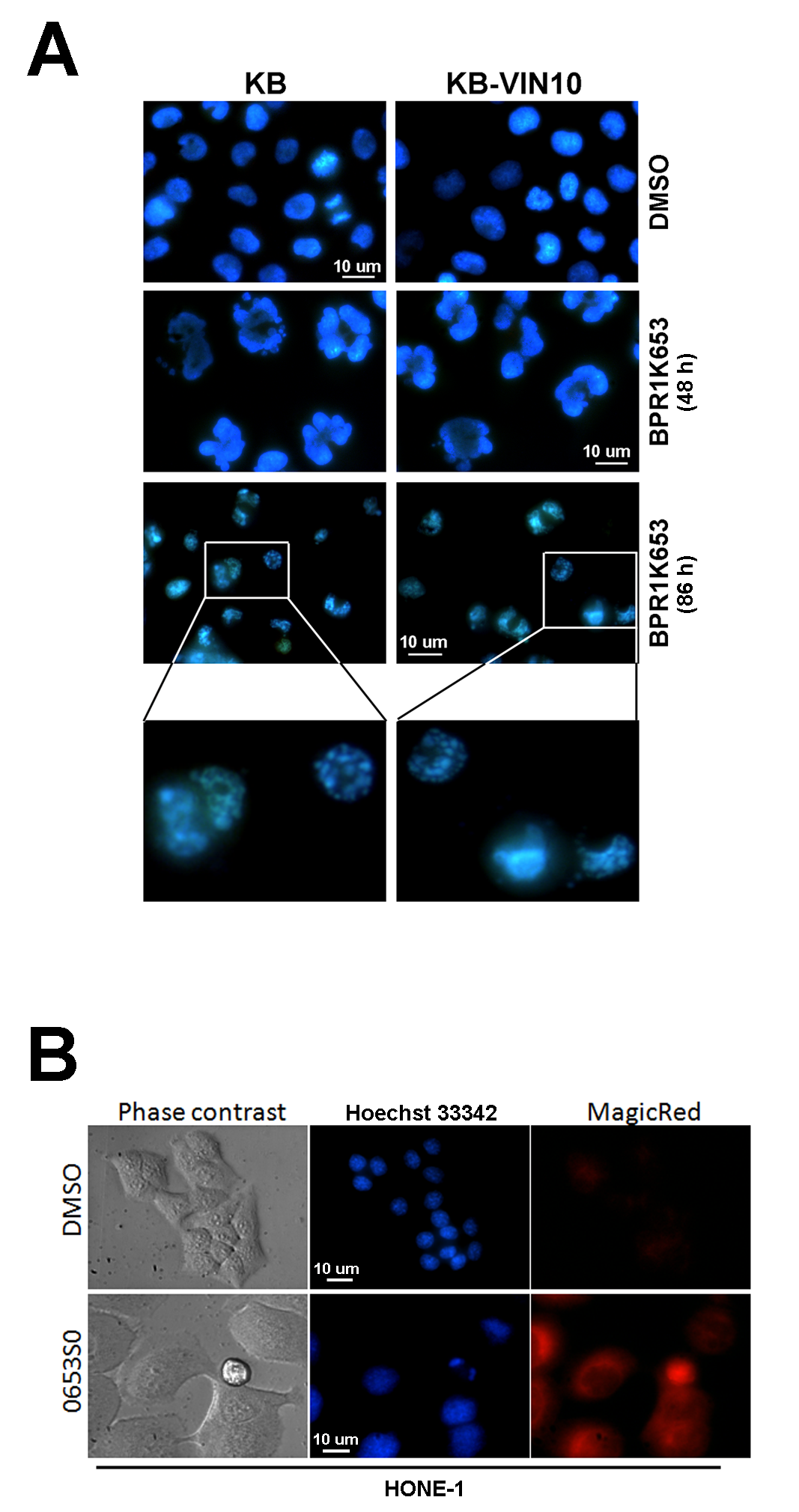

Supplement: Figure S1 — BPR1K653 induces cell endo-replication and apoptosis. (A) BPR1K653 induces endo-replication and subsequent DNA fragmentation in both KB and KB-VIN10 cells. Cells were treated with either DMSO or BPR1K653 for various durations, and nucleus was stained with Hoechst 33342. (B) BRP1K653 induces caspase-3/-7 activity in HONE-1 cancer cells. Cells were treated with either BPR1K653 for 60 h and MagicRed™-DEVD Real-time Caspase-3/-7 Activity kit (Immunochemistry Technologies LLC) was used to detect the activation of caspase-3/-7 in cells, as indicated by the red fluorescent emission. Nucleus was counter-stained blue by Hoechst 33342, and cells were viewed real-time using an UV-enabled inverted microscope. General cell morphology was visualized by phase-contrast microscopy. (TIF) [file pone.0023485.s001.tif]

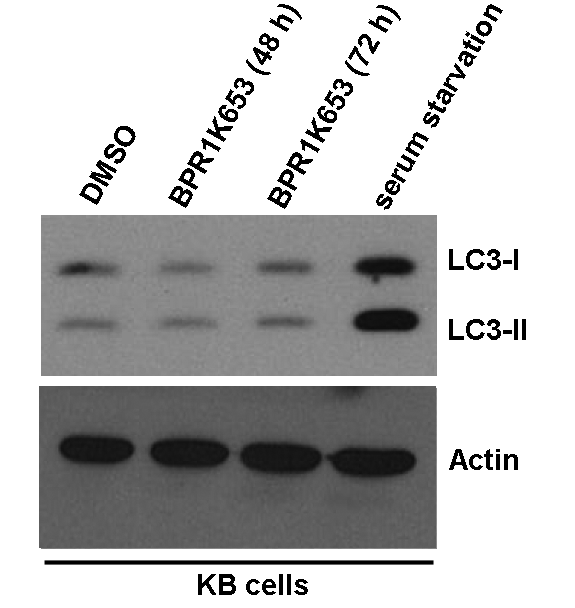

Supplement: Figure S2 — BPR1K653 did not interfere with the process of autophagy in cancer cells. KB cells were treated with either DMSO (negative control) or BPR1K653 (48 h or 72 h) under full serum conditions. Cells cultured drug-free under reduced serum conditions were used as a positive control. Expression of various proteins was determined by Western blotting. The level of conversion of LC3-I to LC3-II provides an indicator of autophagic activity. (TIF) [file pone.0023485.s002.tif]
