# Supplementary material for: BPR1K653, a Novel Aurora Kinase Inhibitor, Exhibits Potent Anti-Proliferative Activity in MDR1 (P-gp170)-Mediated Multidrug-Resistant Cancer Cells
Source: PLoS One. 2011 Aug 24;6(8):e23485. doi: 10.1371/journal.pone.0023485 (PMC3160846; doi:10.1371/journal.pone.0023485)
Supplement: Figure S3 — Details of the composition of the reaction buffers used in different kinase inhibition assay. (DOC) [file pone.0023485.s003.doc]

**Supplemental data 1**

**Different reaction buffers used in the kinase inhibition assay**

**Aurora-A and Aurora-B**

50 mM Tris-HCl pH 7.4, 10 mM NaCl, 10 mM MgCl_2_, 0.01% BSA, 5 mM ATP, 1 mM DTT and 15 mM tetra(LRRASLG) peptide, and either 150 ng recombinant Aurora-A or 40 ng recombinant Aurora-B.

**ALK**

25 mM HEPES pH 7.4, 10 mM MgCl_2_, 4 mM MnCl_2_, 0.5 mM Na_3_VO_4_, 2 mM DTT, 0.02% Triton X100, 0.01% BSA, 1 μM ATP, 2 μM polyGlu4:Tyr peptide, 50~100 ng recombinant ALK.

**CHK**

25 mM HEPES pH 7.4, 10mM MgCl_2_, 4mM MnCl_2_, 0.5mM Na_3_VO_4_, 2 mM DTT, 0.02% Triton X100, 0.01% BSA, 1 mM ATP, 2 mM CHK peptide (KKKVSRSGLYRSPSMPENLNRPR), 50~500 ng recombinant CHK1 or CHK2.

**cMET**

25 mM HEPES pH 7.4, 10mM MgCl_2_, 4 mM MnCl_2_, 0.5mM Na_3_VO_4_, 2 mM DTT, 0.02% Triton X100, 0.01% BSA, 1 mM ATP, 2 mM polyGlu4:Tyr peptide, 75 ng recombinant Met.

**EGFR**

25 mM Tris pH 7.4 (EGFR), 10 m M MgCl_2_, 4 mM MnCl_2_, 0.5 mM Na_3_VO_4_, 2 mM DTT, 0.02% Triton X100, 0.01% BSA, 10 mM ATP, 2 mM polyGlu4:Tyr peptide, 80~500 ng recombinant EGFR.

**FLT3**

75 ng GST-FLT3-KDWT proteins, 25 mM HEPES, pH 7.4, 4 mM MnCl_2_, 10 mM MgCl_2_, 2mM DTT, 0.02% Triton X-100, 0.1 mg/ml bovine serum albumin, 25 μM Her2 peptide substrate, 0.5 mM Na3VO4, and 1 μM ATP.

**VEGFR1/2**

25 mM HEPES pH 7.4, 10 mM MgCl_2_, 4mM MnCl_2_, 0.5mM Na_3_VO_4_, 2 mM DTT, 0.02% Triton X100, 0.01% BSA, 1 mM ATP, 2 mM polyGlu4:Tyr peptide, 50~100 ng recombinant VEGFR1 or VEGFR2.
